# Macroscopic transition metal dichalcogenides monolayers with uniformly high optical quality

**DOI:** 10.1038/s41467-023-37500-1

**Published:** 2023-04-01

**Authors:** Qiuyang Li, Adam Alfrey, Jiaqi Hu, Nathanial Lydick, Eunice Paik, Bin Liu, Haiping Sun, Yang Lu, Ruoyu Wang, Stephen Forrest, Hui Deng

**Affiliations:** 1 Department of Physics, University of Michigan, Ann Arbor, MI 48109 USA; 2 Applied Physics Program, University of Michigan, Ann Arbor, MI 48109 USA; 3 Department of Electrical Engineering and Computer Science, University of Michigan, Ann Arbor, MI 48109 USA; 4 Michigan Center for Materials Characterization, College of Engineering, University of Michigan, Ann Arbor, MI 48109 USA

**Keywords:** Photonic crystals, Nanophotonics and plasmonics, Two-dimensional materials, Polaritons

## Abstract

The unique optical properties of transition metal dichalcogenide (TMD) monolayers have attracted significant attention for both photonics applications and fundamental studies of low-dimensional systems. TMD monolayers of high optical quality, however, have been limited to micron-sized flakes produced by low-throughput and labour-intensive processes, whereas large-area films are often affected by surface defects and large inhomogeneity. Here we report a rapid and reliable method to synthesize macroscopic-scale TMD monolayers of uniform, high optical quality. Using 1-dodecanol encapsulation combined with gold-tape-assisted exfoliation, we obtain monolayers with lateral size > 1 mm, exhibiting exciton energy, linewidth, and quantum yield uniform over the whole area and close to those of high-quality micron-sized flakes. We tentatively associate the role of the two molecular encapsulating layers as isolating the TMD from the substrate and passivating the chalcogen vacancies, respectively. We demonstrate the utility of our encapsulated monolayers by scalable integration with an array of photonic crystal cavities, creating polariton arrays with enhanced light-matter coupling strength. This work provides a pathway to achieving high-quality two-dimensional materials over large areas, enabling research and technology development beyond individual micron-sized devices.

## Abstract (web summary)

The optical quality of large-area transition metal dichalcogenide (TMD) monolayers is usually limited by surface defects and inhomogeneities. Here, the authors report a method based on 1-dodecanol encapsulation to improve the optical properties of TMD monolayers over mm-scale, enabling the fabrication of an array of polariton photonic crystal cavities.

## Introduction

TMD monolayers of composition MX_2_ (M = Mo, W and X = S, Se) form a class of semiconductor that promises novel phenomena and device concepts based on their unique optical properties and their flexibility for engineering and integration^1–3^. However, TMD monolayers created and passivated by existing methods are limited to either micron-scale areas or relatively poor optical qualities, posing severe limitations on further study and developments. For example, light-matter coupling in TMD-based polaritonic or photonic devices are of broad interests recently due to the emerging many-body physics and quantum phenomena^4–8^. However, there are three main limitations from the small size and optical quality of the TMD flakes. First, the long-range coherence, correlation, and transport are limited by the size of the TMD flake, which is usually tens of microns. Second, it is not practical to tune the photonic parameters because a single flake is only able to couple with one (or two for maximum) cavity or photonic crystal. It is reasonably easy to fabricate an array of photonic crystals (PCs) with different parameters, however, it requires transfer of TMD flakes one by one to each of these PCs, which is labor-intensive and highly to be contaminated. Last, optical quality of small TMD flake varies among samples, making the reproduction of experimental results and application challenging. Therefore, a macroscopic TMD monolayer with uniformly high optical quality is highly desired.

The challenge is two-fold, in both the quality and the size of the monolayers. High-quality TMD monolayers can be obtained but mainly are of micron-scale, with encapsulation by hexagonal boron nitride (hBN), where both the TMD and hBN are mechanically exfoliated from bulk crystals using scotch tape or polymers. The exfoliation process is labor intensive and low throughput, producing flakes of random thickness, with only a small fraction being monolayers, with sizes a few to few tens of microns across^9^. Alternative to using exfoliated hBN, other methods have been developed to improve the quantum yield of TMD monolayers, including superacid or ionic salt treatments^10,11^, other chemical passivation methods^12–14^, and passivation by additional layers grown over TMDs^15,16^. While these methods could be compatible with scalable fabrication, their effectiveness has only been shown over micron-scale areas so far.

To obtain larger area monolayers, epitaxial growth^17,18^ and exfoliation by metal tapes^19–21^ have been developed. Yet epitaxial TMD monolayers typically are polycrystalline, have high defect densities, large spatial inhomogeneities, and broad emission linewidths^17,18^. Monolayers exfoliated by gold tapes can reach the centimeter-scale^19–21^, but they suffer from broad exciton linewidth, poor quantum efficiency and large inhomogeneity across the sample; high optical qualities have been reported only when these monolayers were broken into micron-sized flakes and encapsulated by micron-sized exfoliated hBN^19^. The lack of effective methods to encapsulate or passivate these large-area monolayers have limited their usefulness. To date, large-area, high quality TMDs have not been reliably produced.

Here we demonstrate a rapid and reliable method to obtain high-quality TMD macroscopic monolayers (MMLs) of the millimeter to centimeter scales. We show that, while the bare monolayers produced by gold-tape exfoliation showed poor optical quality, full-encapsulation by 1-dodecanol molecular layers lead to significantly improved optical qualities. The resulting encapsulated MMLs exhibit uniform exciton linewidths and quantum yield over the full area of ~3 mm^2^, with values comparable to the best micron-scale monolayers (${{{{{\rm{\mu }}}}}}$MLs) we have obtained. Comparative studies of the optical properties and x-ray photoelectron spectroscopy (XPS) show evidence that the 1-dodecanol layers both passivate chalcogen vacancies in TMDs and suppress substrate quenching of TMD excitons. We furthermore demonstrate that the high optical quality and large size of our MML uniquely allow for scalable integration with an array of many different photonic cavities in the strong coupling regime, with enhanced vacuum Rabi splitting energies. Such high quality MML may enable studies of long range correlations and transport in TMD systems^22–24^ and the development of TMD-based optoelectronic^25^, valleytronic^26^, and quantum devices^27^.

## Results

### Molecular encapsulation of MML

**Fig. 1:**
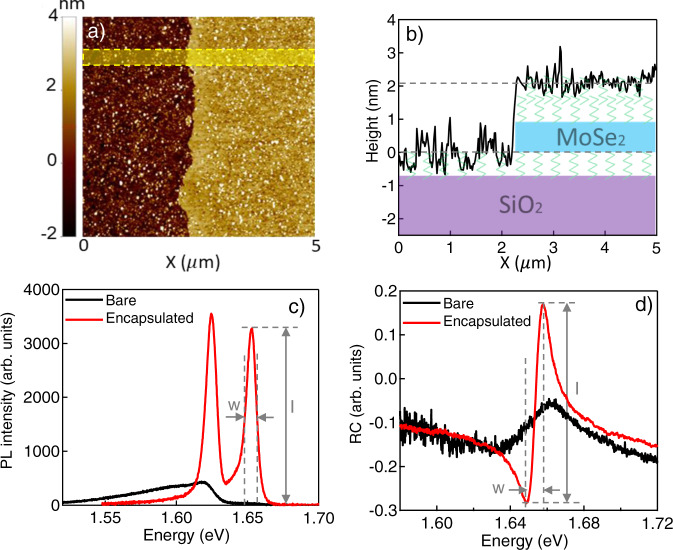
Molecular encapsulation of transition metal dichalcongenides (TMD) macroscopic monolayer (MML) for optical property enhancement. **a** Atomic force microscopy (AFM) image of D/MoSe_2_/D MML over a 5$\times$5 µm^2^ area. **b** The height of the sample surface measured by AFM along the x-axis and averaged over the yellow shaded region marked in (**a**), superposed over a schematic of the sample consisting of, from bottom to top, SiO_2_ substrate, bottom dodecanol layer, MoSe_2_ MML, and top dodecanol layer. **c** Photoluminescence (PL) and (**d**) reflection contrast (RC) spectra of bare (black) and dodecanol-encapsulated (red) MoSe_2_ MML at 5 K. Grey dashed lines mark the height (I) and linewidth (w) of the exciton peak in the PL and RC spectra. For PL, w is the full width at the half-maximum (FWHM).

Figure 1a, b show an atomic force microscopy (AFM) image and a schematic of the molecular-encapsulated MML TMD (D/MoSe_2_/D). To fabricate the sample, we place a self-assembled monolayer of 1-dodecanol on the SiO_2_ substrate. This makes the substrate hydrophobic, as confirmed by water contact angle tests (Supplementary Fig. 1 in Supplementary Information)^28^. Then, we use the previously reported gold-tape exfoliation method to transfer a TMD MML to the dodecanol-passivated surface^19^. Finally, 1-dodecanol layers are drop-cast on the TMD to complete the encapsulation. The top-encapsulation is confirmed by AFM measurements in Fig. 1a. From the dodecanol-covered substrate to the dodecanol-covered MML (along the x-axis and averaged over the yellow shaded region in Fig. 1a), the thickness of the sample increases by 2.1$\pm$0.2 nm (Fig. 1b), corresponding to the sum of the thickness of a MoSe_2_ monolayer (~0.8 nm)^29^ and the dodecanol layer (Supplementary Fig. 2). More details of the fabrication and measurements are described in Methods.

### Optical properties of molecularly encapsulated MML

**Fig. 2:**
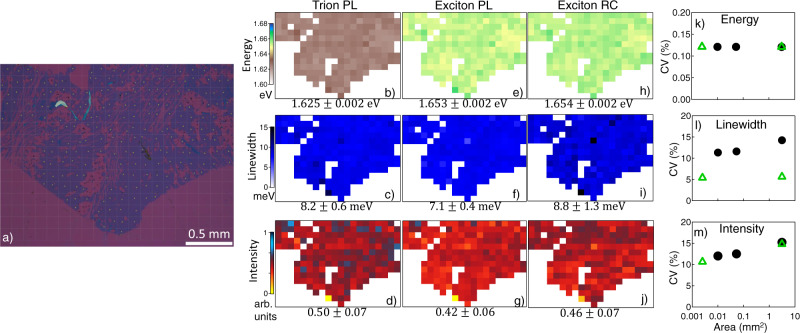
Uniformity of the optical properties over mm-scale. **a** Optical microscope image of a D/MoSe_2_/D MML, where the field of view of 2.500$\times$1.875 mm^2^ is divided into a 20$\times$15 grid of 125 × 125 $\mu$m^2^ square pixels as marked by the grey lines, and PL and RC are measured at a spot (marked by the yellow dots) in each pixel, where there are no visible microscopic wrinkles or cracks. **b**–**j** Maps of the peak energy (top row, **b**, **e**, **h**), linewidth (middle row, **c**, **f**, **i**), and intensity (bottom row, **d**, **g**, **j**) obtained from trion PL (left, **b**–**d**), exciton PL (middle, **e**–**g**), and exciton RC (right, **h**–**j**) spectra. **k**–**m** The coefficient of variation, CV, of (**k**) peak energy, (**l**) linewidth, and (**m**) intensity of exciton PL (green triangle) and RC (black dot) as a function of mapping area.

We characterize the optical qualities of the MML by measuring its exciton spectral weight, linewidth, and quantum yield via reflectance contrast (RC) and photoluminescence (PL) measurements and compare the encapsulated MoSe_2_ MMLs with ones without encapsulation. Fig. 1c, d show representative examples of PL and RC spectra at 5 K from randomly chosen spots on the sample. Spatial distributions of the optical properties over the entire MML are shown in Fig. 2 and Supplementary Figs. 8, 9.

The PL spectra in Fig. 1c shows sharp contrast between the bare and encapsulated MoSe_2_ MML. The bare MML PL is dominated by broad in-gap state (IGS) emission, spreading from ~1.60 eV to ~1.63 eV^30^. The encapsulated MML, however, features two sharp peaks at 1.653 and 1.625 eV, with full width at half maximum (FWHM) of 7.1$\pm$0.4 and 8.2$\pm$0.6 meV, respectively, corresponding to MoSe_2_ monolayer A exciton and trion emission^30^, while IGS emission below the excitonic resonances is strongly suppressed.

By comparing the integrated PL intensity with a reference sample, we obtain the PL quantum yield (PLQY) of the samples (see Methods for details). With dodecanol encapsulation, the total PLQY is increased by nearly 3-fold, from 6.2% to 18%, while the PLQY integrated over the exciton and trion resonances (1.60 eV–1.68 eV) is increased by 7-fold.

In the RC spectra (Fig. 1d), the exciton linewidth is sharpened from 26$\pm$3 to 8.8$\pm$1.3 meV after encapsulation, showing considerably improved spatial homogeneity. At the same time, the exciton peak height (${{{{{\rm{I}}}}}}$, marked in gray line) is increased by 4-fold after encapsulation, indicating an enhanced oscillator strength and radiative recombination rate of exciton state^31^, consistent with suppressed IGS emission.

The above studies show that, while the emission of bare MoSe_2_ MML is overwhelmed by IGS and has significant inhomogeneities, dodecanol encapsulation largely eliminates IGS and restores the intrinsic excitonic features of the MoSe_2_ monolayers. The resulting optical quality of the encapsulated MML, measured by the exciton and trion linewidths, spectral weight and PLQY, is close to that of the best hBN-encapsulated ${{{{{\rm{\mu }}}}}}$MLs we can obtain from the same commercial crystals (see Supplementary Fig. 4a, b and Supplementary Table 1). We have also compared the dodecanol encapsulation with acid^10^ or ionic salt^11^ treatments that have been shown to enhance the PLQY for ${{{{{\rm{\mu }}}}}}$MLs. When applied to our MMLs, the treatments mainly enhanced the IGS and trion PLQY at 5 K (see Methods and Supplementary Fig. 4c).

To test the stability of the encapsulated MML, we monitored the PL spectra at the same location of a D/MoSe_2_/D MML sample and found that the high optical quality of the MML can be maintained over extended periods of time (90 days) by reapplication of dodecanol to exposed surfaces (Supplementary Fig. 5). Moreover, dodecanol encapsulation is also effective in passivating WS_2_ MML and on a variety of substrates, including the widely used fused silica and silicon nitride (Supplementary Fig. 6).

### Uniformity of the optical properties

While the foregoing results focus on optical properties at specific spots on the sample, a key metric for large-area samples is their uniformity. Even in ${{{{{\rm{\mu }}}}}}$MLs, there is often significant variation of the optical properties from spot to spot within the same flake and from one flake to another, due to disorders and strain introduced during exfoliation and transfer^17^. To quantify the uniformity of our D/MoSe_2_/D MMLs, we mapped the peak energy, linewidth, and intensity of the exciton and trion resonances in both PL and RC across the MML, as shown in Fig. 2. The spatial resolution of the PL and RC measurements are 2.5 and 23 ${{{{{\rm{\mu }}}}}}$m, corresponding to the diameter of the focused spot of the pump laser and white light used, respectively. For each map, we obtain the average value, standard deviation, and coefficient of variation (CV = standard deviation/average value) of the corresponding property, as listed below the map.

The D/MoSe_2_/D MML shows remarkable uniformity in both PL and RC spectra, where the exciton and trion energies have a standard deviation of 2 meV (CV = 0.1%), and their linewidths vary by 0.4–0.6 meV (CV = 6–7%). From the difference of the exciton and trion energies, we obtain the trion binding energy of 28.3$\pm$0.6 meV (Supplementary Fig. 7a), consistent with previously reported values^30^. The PL intensities of the exciton and trion both vary by 14%, and their ratio is 0.84$\pm$0.10 (Supplementary Fig. 7b). Their sum gives PLQY = 15%$\pm$2%, with a maximum PLQY = 22%.

We have furthermore performed continuous spatial mapping over 50$\times$50 µm^2^, 100$\times$100 µm^2^, and 230$\times$230 µm^2^ areas on the sample_,_ all with spot-size limited resolution (Supplementary Figs. 8, 9). Fig. 2k–m show CVs of the exciton energy, linewidth, and intensity as a function of the mapping area. The CVs barely change in both RC (black dots) and PL (green triangles) even as the mapping area is increased by over 3 orders of magnitude. This suggests that every 2.5$\times$2.5 ${{{{{\rm{\mu }}}}}}$m^2^ area of the mm-scale D/MoSe_2_/D MML has nearly identical optical properties, with the same exciton energy, linewidth, and PLQY.

### Proposed mechanisms of optical property enhancement

**Fig. 3:**
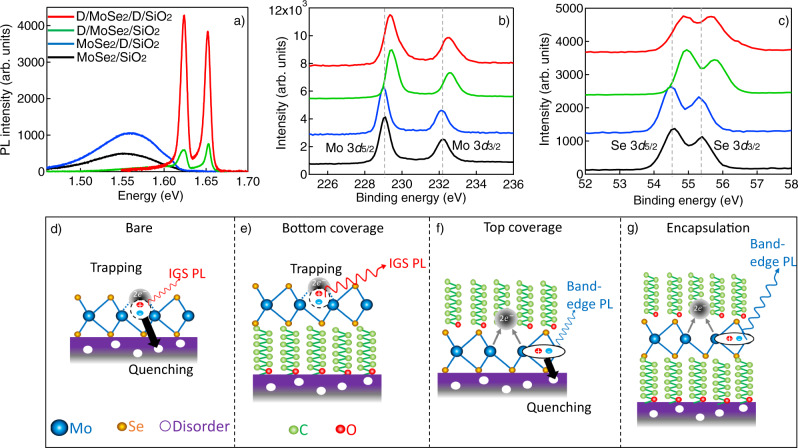
Proposed mechanisms of optical property enhancement due to dodecanol encapsulation. **a** Comparison of PL spectra of bare (black), only bottom-covered (blue), only top-covered (green), and both top and bottom-covered (red) MoSe_2_ MML. **b**, **c** Comparison of X-ray photoelectron spectroscopy (XPS) spectra of (**b**) Mo 3*d* core levels and (**c**) Se 3*d* core levels of the same four MMLs as in (**a**), showing blueshifts when there is top-coverage of dedocanol (green and red). **d**–**g** Illustration of the effects of dodecanol on the MoSe_2_ MML optical properties. **d** Bare MoSe_2_ MML suffers from in-gap state (IGS) emission (small red wavy arrow) due to exciton trapped at Se vacancies and PL quenching (black arrow) due to disorders in substrate. **e** Bottom dodecanol layer separates MoSe_2_ from the substrate. **f** Top dodecanol layer passivates Se vacancies and leads to band-edge PL emission (blue wavy arrow). **g** Top and bottom layers complete the encapsulation.

To understand the mechanisms of optical property enhancement by dodecanol encapsulation, we compare the PL and X-ray photoelectron spectroscopy (XPS) spectra of MMLs without and with different dodecanol coverages, including a bare MML (MoSe_2_/SiO_2_), with dodecanol on the bottom surface only (MoSe_2_/D/SiO_2_), on the top surface only (D/MoSe_2_/SiO_2_), and on both top and bottom surfaces (D/MoSe_2_/D/SiO_2_), as shown in Fig. 3.

The PL spectra (Fig. 3a) reveal that bottom and top dodecanol coverages have distinctly different effects. The bottom layer leads to enhanced emission intensities for the IGS-dominated spectra but no change in the spectral distribution; in contrast, the top layer leads to similar overall emission intensities but greatly suppressed IGS emission.

With the bottom layer only, the spectral distribution remains the same, which suggests that the bottom layer does not change the electronic states of the MML. However, the PL intensity is significantly enhanced for both IGS and excitonic states. One possible explanation is that the bottom layer isolates the MML from charge disorders on the substrate surface, thereby suppressing substrate-induced non-radiative decay, leading to enhanced PL intensity, similar to how hBN encapsulation improves TMD quantum yield^17,32^.

In contrast, the top dodecanol layer leads to a drastically different spectrum but no significant change in the total emission intensity. Without top encapsulation (MoSe_2_/SiO_2_ and MoSe_2_/D/SiO_2_), the spectra are dominated by IGS emission; with top encapsulation (D/MoSe_2_/SiO_2_ and D/MoSe_2_/D/SiO_2_) the emission is dominated by excitons and trions with negligible IGS emission. This suggests that the top dodecanol layer effectively removes IGS, most likely by passivating the impurities in the MML.

To further verify passivation of IGS, we measure whether the electronic states in the MML have been modified via XPS. We compare the XPS spectra (Fig. 3b, c) of Mo and Se 3*d* core levels of the four MoSe_2_ MMLs. For bare MoSe_2_/SiO_2_ and bottom-covered MoSe_2_/D/SiO_2_ MMLs, the 3*d*_5/2_ and 3*d*_3/2_ core level peaks of Mo (229.2$\pm$0.1 and 232.3$\pm$0.1 eV) and Se (54.5$\pm$0.1 and 55.4$\pm$0.1 eV) are consistent with values reported in the literature^33^. However, for the top-covered D/MoSe_2_/SiO_2_ and D/MoSe_2_/D/SiO_2_, both Mo and Se 3*d* core level peaks are upshifted by 0.4$\pm$0.1 eV, suggesting an increased ionization energy of a 3*d* core level electrons in Mo and Se. Since the MML is not grounded, this increased ionization energy can be understood as a result of electron transfer from MoSe_2_ to the top-covered 1-dodecanol, which reduces the electron density in Mo and Se outer shells and enhances the attractive interaction in the inner shell (3*d*)^34^.

Based on the above observations, we propose the following passivation mechanism by the top dodecanol layer (Fig. 3d–g). As shown in previous studies, the negatively charged chalcogen vacancies that serve as hole traps are the dominant surface defects in exfoliated TMDs^35,36^. The photogenerated excitons are trapped at the Se vacancies in MoSe_2_ and recombine as IGS emission. XPS data show that the top dodecanol efficiently promotes electron transfer from MoSe_2_, which removes the accumulated negative charges at the vacancies, leading to enhanced free exciton and trion emission. Only the top dodecanol layer induces efficient charge transfer. This may be due to the orientation of the 1-dodecanol relative to the MoSe_2_ MML. In the bottom dodecanol layer on SiO_2_, the OH functional group in dodecanol reacts with Si to form Si-O bonds^37^, leaving the C-chain facing the MoSe_2_ MML. On the other hand, in the top dodecanol, it is likely that the OH functional group faces the MoSe_2_ to induce the effective charge transfer.

The bottom dodecanol, however, is also important. Without it, the quenching due to SiO_2_ surface defects and patch charge remains, irrespective of the passivation, leading to low PLQY for both IGS and excitonic states. With top and bottom encapsulation together (D/MoSe_2_/D), the TMD is protected from both the IGS and the disorders from substrates.

Further studies of surface chemistry, such as through scanning tunneling microscopy, may allow direct verification of the microscopic mechanism of passivation.

### Scalable integration and strong coupling of MML-PC arrays

**Fig. 4:**
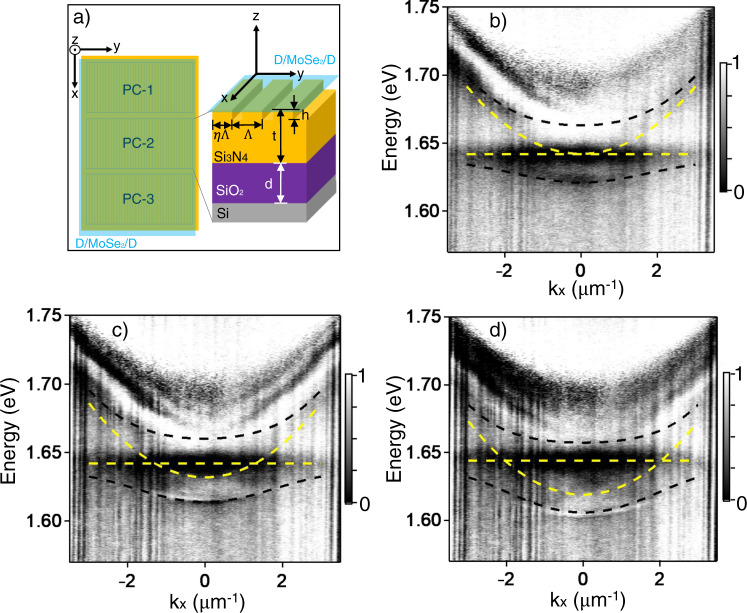
Strong coupling between D/MoSe_2_/D MML excitons and 1D photonic crystals (PCs). **a** Schematic of three 1D silicon nitride PCs (yellow) with a D/MoSe_2_/D MML placed on top (blue). Each PC is 200$\times$100 ${{{{{\rm{\mu }}}}}}$m^2^ in size and has about the same filling factor of *η*, total thickness of t, grating thickness of h, on the top of a SiO_2_ layer with a thickness of d, but varying in the period Λ to achieve different cavity detuning. **b**–**d** Angle-resolved RC data of three D/MoSe_2_/D-PC integrated devices on the same chip, with the PC mode detuning of (**b**) 0.1$\pm$0.2 meV, (**c**) 10$\pm$1 meV, and (**d**) 23$\pm$1 meV. Black dashed lines mark the lower and upper polariton bands. Yellow dashed lines mark the uncoupled bare exciton and PC resonances.

To illustrate the utility of the high-quality MMLs for integrated photonic devices, we demonstrate strong coupling of one MML with many different PCs on a millimeter-sized chip. This is not possible with ${{{{{\rm{\mu }}}}}}$MLs, as each $\mu$ML can only cover one PC after careful alignment, while additional transfer would produce cross-contamination of the ${{{{{\rm{\mu }}}}}}$MLs. The MML, however, readily covers a large array of PCs of varying parameters as illustrated in Fig. 4a.

Details of the PC structure and its fabrication process are described in Methods and in Refs. ^38,39^. The PC is anisotropic and we utilize the transverse-electric (TE) polarized modes with electric field along the grating bar, x-direction. The energy of the PC modes is tuned by the grating period (Λ). Here we use three PCs, PC-1, 2, and 3, with $\Lambda=$ 491, 495, and 499 nm, respectively. Supplementary Fig. 10a–c show the momentum-resolved RC of the bare PCs, where we observe resonant modes with parabolic dispersions. From their FWHM (w_cav_=1.0$\pm$0.2 meV, Supplementary Fig. 10d), the quality factor (Q) is estimated to be > 600 for all three PCs.

After placing the D/MoSe_2_/D MML onto the gratings (Supplementary Fig. 10e), we observe two polariton bands (marked with black dashed lines in Fig. 4b–d) in the RC spectrum of each device, showing strong coupling between the MML MoSe_2_ exciton and PC modes. To confirm strong coupling, we fit the measured LP and UP dispersions, ${{{{{{\rm{E}}}}}}}_{{{{{{\rm{LP}}}}}},{{{{{\rm{UP}}}}}}}({{{{{{\rm{k}}}}}}}_{{{{{{\rm{x}}}}}}})$, to extract the coupling strength (g) and Rabi splitting (2ħΩ) with:

1 $${{{{{{\rm{E}}}}}}}_{{{{{{\rm{LP}}}}}},{{{{{\rm{UP}}}}}}}({k}_{x})=	\frac{1}{2}\left[{{{{{{\rm{E}}}}}}}_{{{{{{\rm{exc}}}}}}}+{{{{{{\rm{E}}}}}}}_{{{{{{\rm{cav}}}}}}}({k}_{x})+{{{{{\rm{i}}}}}}\frac{\left({{{{{{\rm{w}}}}}}}_{{{{{{\rm{cav}}}}}}}+{{{{{{\rm{w}}}}}}}_{{{{{{\rm{exc}}}}}}}\right)}{4}\right]\\ 	 \pm \sqrt{{{{{{{\rm{g}}}}}}}^{2}+\frac{{\left[{{{{{{\rm{E}}}}}}}_{{{{{{\rm{exc}}}}}}}-{{{{{{\rm{E}}}}}}}_{{{{{{\rm{cav}}}}}}}({k}_{x})+{{{{{\rm{i}}}}}}\left({{{{{{\rm{w}}}}}}}_{{{{{{\rm{cav}}}}}}}-{{{{{{\rm{w}}}}}}}_{{{{{{\rm{exc}}}}}}}\right)/2\right]}^{2}}{4}}$$

2 $$2\hslash \varOmega=\sqrt{4{{{{{{\rm{g}}}}}}}^{2}-{({{{{{{\rm{w}}}}}}}_{{{{{{\rm{cav}}}}}}}-{{{{{{\rm{w}}}}}}}_{{{{{{\rm{exe}}}}}}})}^{2}/4}$$

where E_exc_ and E_cav_ are the uncoupled exciton energy and cavity mode energy, respectively; w_exc_ and w_cav_ are FWHM of the uncoupled exciton and PC resonance mode, respectively. ${{{{{{\rm{E}}}}}}}_{{{{{{\rm{cav}}}}}}}\left({k}_{x}\right)$ is obtained by fitting the bare cavity dispersion to a parabola (Supplementary Fig. 10a–c, black dashed lines), and we directly measure E_exc_ = 1.642$\pm$0.002 eV and w_exc_ = 18$\pm$1 meV from RC and PL of D/MoSe_2_/D MML on the same device outside the PC region (Supplementary Fig. 6a). Both E_LP_(*k*_*x*_) and E_UP_(*k*_*x*_) of all 3 devices are fit globally (black dashed lines in Fig. 4b–d), yielding g = 23$\pm$2 meV and 2; ħΩ = 43 ± 3 meV. These values satisfy the strong coupling condition:

3 $$2\hslash \varOmega \, > \, ({{{{{{\rm{w}}}}}}}_{{{{{{\rm{cav}}}}}}}+{{{{{{\rm{w}}}}}}}_{{{{{{\rm{exc}}}}}}})/2=10\,{{{{{\rm{meV}}}}}},\, {{{{{\rm{2g}}}}}} \, > \, \sqrt{\frac{{{{{{{\rm{w}}}}}}}_{{{{{{\rm{cav}}}}}}}^{2}+{{{{{{\rm{w}}}}}}}_{{{{{{\rm{exc}}}}}}}^{2}}{2}}=12\,{{{{{\rm{meV}}}}}}$$

which confirms the D/MoSe_2_/D-PC system is well into the strong coupling regime.

The measured coupling strength or Rabi splitting is more than twice as large as previously reported for bare TMD $\mu$MLs on a similar PC^39^ and over 30% larger than that of hBN-covered^5^ and hBN-encapsulated^7,8^ MoSe_2_ ${{{{{\rm{\mu }}}}}}$MLs in a distributed Bragg reflector-based cavity. The large coupling strength suggests enhanced oscillator strength due to passivated defects (vacancies) of our encapsulated MML^31^. Besides the enhanced light-matter coupling strength, compared with ${{{{{\rm{\mu }}}}}}$ML flake-based polaritonic devices, our work shows two main improvements: first, the uniform large area of MML enables studies of long-range coherence and transport of exciton-polaritons, no longer limited to $\mu$m-scale size; second, it allows excitons from the same MML with uniform properties to couple with an arrays of photonic devices with tunable photonic parameters, which cannot be achieved by ${{{{{\rm{\mu }}}}}}$ML flakes.

## Discussion

In summary, we demonstrate a method to create high-quality, macroscopic-sized TMD monolayers via 1-dodecanol encapsulation of gold-exfoliated TMDs. We show that the encapsulating molecular layers both passivate the chalcogen vacancies and suppress non-radiative recombination into the substrate. The exciton and trion linewidths, spectral weights and PLQY are slightly worse but close to hBN-encapsulated $\mu$MLs. Within optical resolution, the excitonic properties are highly uniform across the whole mm-scale sample. The method is fast and can be readily reproduced at a wet bench using commercially available materials. It is also compatible with scalable integration of the TMD with desired substrates and structures, as we illustrate by integration and strong coupling of the MML with an array of cavities for integrated polaritonic devices. These results open a pathway for rapid production of high-quality, large-area 2D van der Waals materials.

## Methods

### Placing dodecanol self-assembled monolayer (SAM) on SiO_2_/Si substrate

We place a self-assembled monolayer of 1-dodecanol on the substrate, which prepares the substrate to be hydrophobic^32,37^. To do so, we drop-cast the 1-dodecanol liquid on the surface of SiO_2_(285 nm)/Si substrate at 180 °C on a hot plate and keep it for 2 min. Then the dodecanol is washed out with isopropanol and blown dry with a nitrogen gun. The hydrophobic surface is characterized by the water contact angle as shown in Supplementary Fig. 1 in Supplementary Information.

### Gold-tape exfoliation of transition metal dichalcogenides (TMD) mm-scale monolayer (MML)

We prepare TMD MMLs following a similar procedure as previously reported^19^. We first deposit a 150 nm gold film on Si wafer by e-beam evaporation (0.05 nm/s), then spin-coat the gold film by polyvinylpyrrolidone (PVP) solution (Sigma Aldrich, mw 40000, 10% wt in ethanol/acetonitrile wt 1/1) at 1500 rpm for 2 min, with the acceleration of 500 rpm/s, and anneal at 150 °C for 2 min. A single-sided heat release tape is cut into small pieces (~1$\times$1 cm^2^ square) and stuck onto the PVP/gold surface to peel off the gold film from the Si wafer to form a gold tape, which is then gently pressed onto a TMD single crystal (purchased from HQ Graphene) to exfoliate a monolayer. The TMD monolayer on the gold tape is then transferred onto a desired substrate. In this work, substrates used include SiO_2_(285 nm)/Si with and without 1-dedaconal on top, fused silica, and silicon nitride substrates. We then heat the substrate with everything on top using a hot plate at 135 °C for 3 min to remove the heat release tape, followed by water soaking for 4 h to remove the PVP layer on gold. Finally, the gold layer is removed by gold etchant (2.5 g I_2_ and 10 g KI in 100 mL deionized water), and the TMD monolayer on substrate is washed by water and isopropanol, then dried by a nitrogen gun.

### Dodecanol encapsulation

We drop-cast 1-dodecanol on the top of as-prepared TMD MML on the substrate at 180 °C on a hot plate and keep it for 2 min. Then the dodecanol was gently washed out by immersion in isopropanol and blew dry with a nitrogen gun. The dodecanol that remains on the surface is verified by atomic force microscopy (AFM) measurement, as shown in Supplementary Fig. 2 for multiple applications of the dodecanol.

### Photonic crystal (PC) and D/MoSe_2_/D-PC device

The PCs used in this work are made of a silicon nitride layer deposited on a SiO_2_/Si substrate by low-pressure chemical vapor deposition^38,39^. The silicon nitride layer is partially etched to form a 1D grating by electron beam lithography followed by plasma dry etching. As shown in Fig. 4a, the total silicon nitride grating thickness (t) is 113 nm, SiO_2_ capping layer thickness (d) is 1475 nm, grating step height (h) is 60 nm, and the gap between grating bars is 50 nm. The grating period (Λ) is 491, 495, and 499 nm for PC-1, PC-2, and PC-3, respectively.

### Acid and ionic salt treatments on MoSe_2_ MML

The bis(trifluoromethane)sulfonimide (TFSI) acid and bis(trifluoromethane)sulfonimide lithium salt (TFSI-Li) treatments are done following similar procedures as reported previously^10,11^. For TFSI/MoSe_2_ MML, 0.02 M TFSI solution (in 1, 2-dichloroethane) is put in a closed vial. MoSe_2_ MML sample is immersed into the TFSI solution inside the vial and heated at 100 °C for 10 min. The sample is dried and annealed on a hot plate at 100 °C for 5 min, ready for use. For TFSI-Li/MoSe_2_ MML, 0.02 M TFSI-Li salt solution (in methanol) is put in a closed vial. MoSe_2_ MML sample is immersed into the TFSI solution inside the vial for 40 min at room temperature. The sample is dried and annealed on a heat plate at 100 °C for 5 min, and is ready for use.

### Optical measurements

RC and PL spectroscopies are conducted by real-space and Fourier-space imaging of the sample/device. An objective lens with numerical aperture (NA) of 0.55 is used for both focusing and collection. Two white light sources are used for RC measurements: a tungsten halogen lamp with a beam size of ~23 μm in diameter and a high-power supercontinuum white light laser with a beam size of ~5 μm in diameter. For PL, a continuous-wave solid-state laser at 532 nm with a power of 30 ${{{{{\rm{\mu }}}}}}$ W and a beam size of ~2.5 ${{{{{\rm{\mu }}}}}}$m in diameter is used to excite TMD samples. The collected signals are detected by a Princeton Instruments spectrometer with a cooled charge-coupled camera. The sample is kept at 5 K using a Montana Fusion system.

### PL quantum yield (PLQY) measurement

PLQY of hexagonal boron nitride (hBN) and dodecanol encapsulated MoSe_2_ samples at 5 K are measured following the previously reported procedures^15^. We use a 50 nm-thick organic film (10% PQIr:CBP) on SiO_2_ (285 nm)/Si substrate as a reference material, which is made of 10% iridium (III) bis(2-phenyl quinolyl-N,C20) acetylacetonate blended in 4,4′-Bis(N-carbazolyl)−1,1′-biphenyl). Its PLQY is determined to be ~81% at 298 K with 532 nm CW laser excitation (50 ${{{{{\rm{\mu }}}}}}$W) using an integrating sphere; the value is repeatable and agrees with what has been reported in the literature^15^. Then we determine the PLQY of TMD samples by comparing TMD samples (at 5 K) and the same reference sample (at 298 K) on the same SiO_2_ (285 nm)/Si substrate with the same confocal microscopy setup and the same 532 nm CW laser excitation power (50 ${{{{{\rm{\mu }}}}}}$ W). The PLQY was calibrated using the integration of their PL spectra from 1.55 to 2.25 eV. The double absorption at the 532 nm excitation wavelength (considering the back reflection from the substrate) are considered in calculating the PLQY. The absorption coefficient for the MoSe_2_ monolayer (thickness of ~0.8 nm) used in the PLQY calculation follows the value reported in Ref. ^40^; while the absorption coefficient for the organic film is determined by the UV-Vis spectrum measured on the same film grown on a transparent fused silica substrate (Supplementary Fig. 3a). The PL spectra of dodecanol-encapsulated MoSe_2_ samples (at 5 K) along with the reference material (at 298 K) are shown in Supplementary Fig. 3b.

### PL and RC mapping

Besides the mm-scale mapping shown in Fig. 2, we have also performed resolution-limited, full-area mapping of both PL and RC over smaller regions, using the beam size (corresponding to the spatial resolution) as the step size. The PL mapping is performed with a 532 nm continuous-wave pump laser with a beam size of ~2.5 ${{{{{\rm{\mu }}}}}}$m, over a region of 50$\times$50 ${{{{{\rm{\mu }}}}}}$m^2^ divided to 20$\times$20 pixels. The results are shown in Supplementary Fig. 8. The RC mapping is performed over two regions – a larger region of 230$\times$230 ${{{{{\rm{\mu }}}}}}$m^2^ in size, divided into 10$\times$10 pixels, using a tungsten light of the focused beam size of ~23 ${{{{{\rm{\mu }}}}}}$m, and a smaller region of 100$\times$100 ${{{{{\rm{\mu }}}}}}{{{{{{\rm{m}}}}}}}^{2}$ in size, divided into 20$\times$20 pixels, using a supercontinuum of the focused beam size of ~5 ${{{{{\rm{\mu }}}}}}$m. The results are shown in Supplementary Fig. 9.

### AFM measurements

All AFM images are taken at ambient conditions with a Park Systems NX10 system using Olympus Cantilever OMCL-AC160TS tips under tapping mode.

### X-ray photoelectron spectroscopy (XPS) measurements

XPS measurements are conducted at room temperature using Kratos Axis Ultra XPS system with a monochromatic Al source.

### Supplementary information

Supplementary Information

Peer Review File

## Data Availability

The raw and source data generated in this study have been deposited in the University of Michigan - Deep Blue Data (10.7302/m1b1-cf17).
