# Author Correction: Macroscopic transition metal dichalcogenides monolayers with uniformly high optical quality

**DOI:** 10.1038/s41467-023-42119-3

**Published:** 2023-10-06

**Authors:** Qiuyang Li, Adam Alfrey, Jiaqi Hu, Nathanial Lydick, Eunice Paik, Bin Liu, Haiping Sun, Yang Lu, Ruoyu Wang, Stephen Forrest, Hui Deng

**Affiliations:** 1 Department of Physics, University of Michigan, Ann Arbor, MI 48109 USA; 2 Applied Physics Program, University of Michigan, Ann Arbor, MI 48109 USA; 3 Department of Electrical Engineering and Computer Science, University of Michigan, Ann Arbor, MI 48109 USA; 4 Michigan Center for Materials Characterization, College of Engineering, University of Michigan, Ann Arbor, MI 48109 USA

**Keywords:** Photonic crystals, Nanophotonics and plasmonics, Two-dimensional materials, Polaritons

Correction to: *Nature Communications* 10.1038/s41467-023-37500-1, published online 01 April 2023

In the “Acknowledgements” section of the original version of this Article, the grant number relating to the Gordon and Betty Moore Foundation was incorrectly given as N031710 and should have been GBMF10694. This has now been corrected in both the PDF and HTML versions of the Article.

