# Supplementary material for: Macroscopic transition metal dichalcogenides monolayers with uniformly high optical quality
Source: Nat Commun. 2023 Apr 1;14:1837. doi: 10.1038/s41467-023-37500-1 (PMC10067954; doi:10.1038/s41467-023-37500-1)
Supplement: Supplementary file 1 — Supplementary Information [file 41467_2023_37500_MOESM1_ESM.pdf]

Supplementary Information for

Macroscopic Transition Metal Dichalcogenides Monolayers with Uniformly High Optical Quality

Qiuyang Li,[1] Adam Alfrey,[1] Jiaqi Hu,[2] Nathanial Lydick,[1] Eunice Paik,[1] Bin Liu,[3] Haiping Sun,[4] Yang Lu,[3] Ruoyu Wang,[1,3] Stephen Forrest,[1,2,3] Hui Deng[1,2,*]

[1]Department of Physics, University of Michigan, Ann Arbor, Michigan 48109, United States

[2]Applied Physics Program, University of Michigan, Ann Arbor, Michigan 48109, United States

[3]Department of Electrical Engineering and Computer Science, University of Michigan, Ann Arbor, Michigan 48109, United States

[4]Michgian Center for Materials Characterization, College of Engineering, University of Michigan, Ann Arbor, Michigan 48109, United States

*Corresponding author. Email: dengh@umich.edu

Content list

Supplementary Figures 1 to 10

Supplementary Table 1

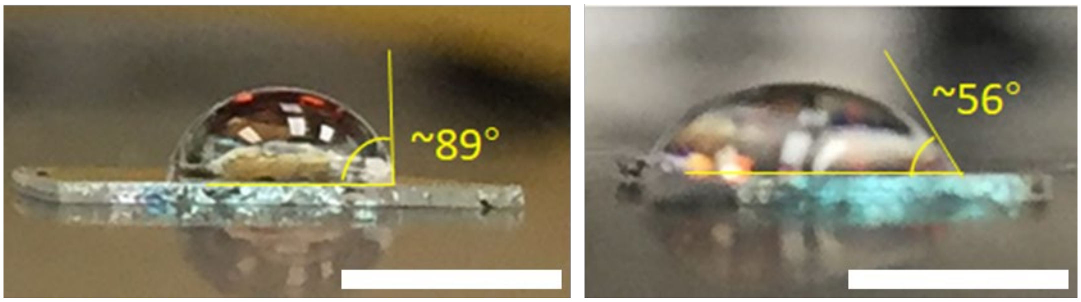

Supplementary Figure 1. Verification of 1-dodecanol coverage of the substrate. Water contact angle on SiO_2 surface with (left) and without (right) 1-dodecanol self-assembled monolayer (SAM). The scale-bar is 5 mm.

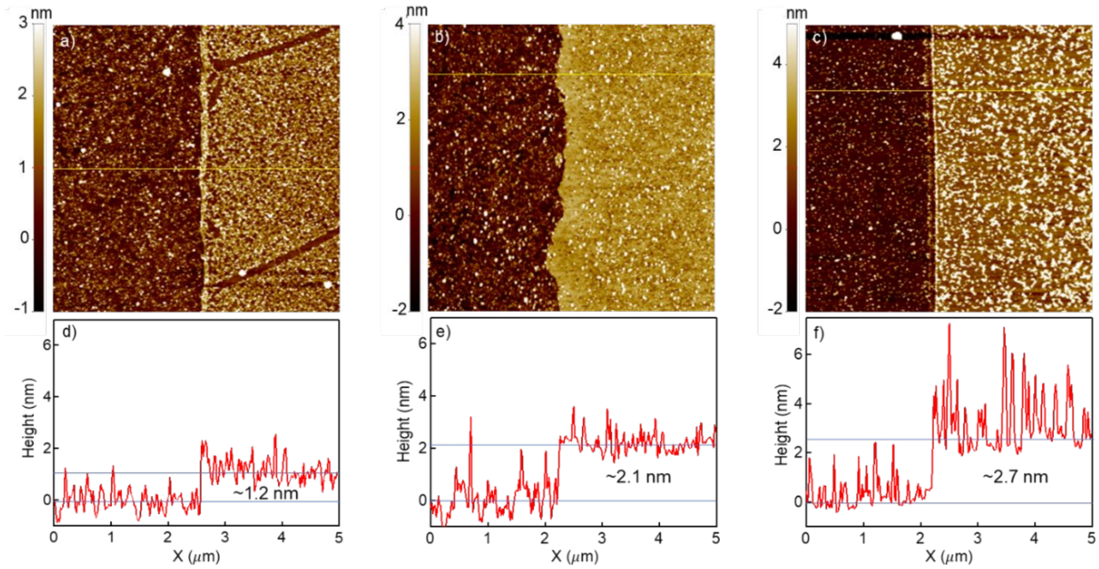

Supplementary Figure 2. Measurements of the dodecanol thickness. Atomic force microscopy (AFM) images of D/$MoSe_2$/D macroscopic monolayer (MML) with top surface treated with dodecanol for (a) once, (b) twice, and (c) 5 times. The height as a function of x-axis along the yellow line in (a-c) is shown in (d-f), respectively.

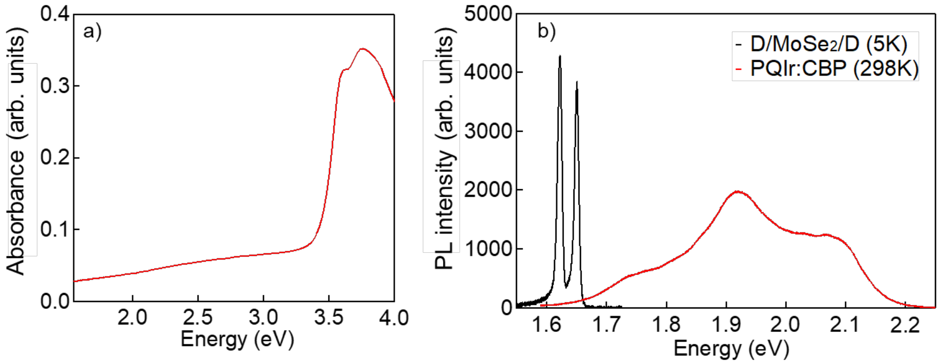

Supplementary Figure 3. Measurements of the photoluminescence quantum yield (PLQY). (a) Absorption spectrum of the 10% PQIr:CBP organic film at 298K. (b) The photoluminescence (PL) spectra of dodecanol-encapsulated $MoSe_2$ MML at 5K (black), and PQIr:CBP film at 298K (red).

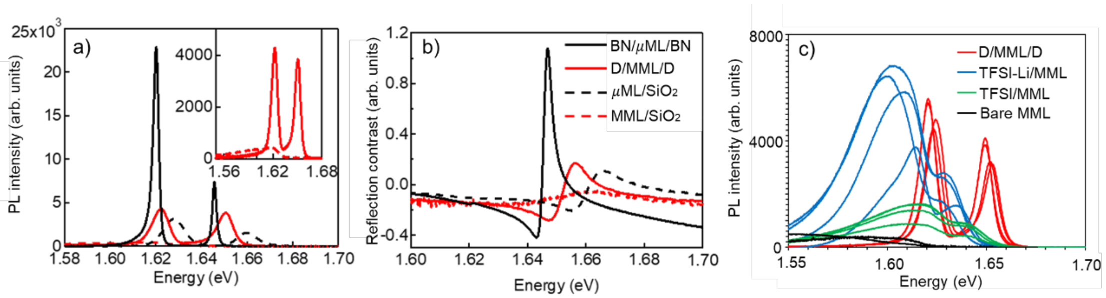

Supplementary Figure 4. Comparison of MMLs, μMLs and different encapsulation and passivation methods. (a) Photoluminescence (PL) and (b) reflection contrast (RC) spectra of MMLs (red lines) and μMLs (black lines) with and without encapsulation (solid and dashed lines) by dodecanol and hBN, respectively. Inset of (a): Zoomed in PL spectra of D/$MoSe_2$-MML/D (red solid line) and bare $MoSe_2$ MML (red dashed line). The bare MML shows very broad linewidth, small spectral weight and weak PL intensity dominated by IGS. The D/$MoSe_2$-MML/D, however, shows comparable qualities as the μMLs – better than bare μML and slightly worse than hBN-encapsulated μML. (c) Comparison of dodecanol encapsulation (red lines) vs. acid and ionic salt treatments (blue and green lines) for MMLs. Shown are PL spectra of several randomly picked spots on dodecanol encapsulated (D/MML/D), bis(trifluoromethane)sulfonimide treated (TFSI/MML), bis(trifluoromethane)sulfonimide lithium salt treated (TFSI-Li/MML), and bare $MoSe_2$ MML. Acid treatments mainly enhance IGS and trion emission. All μMLs and MMLs are exfoliated from the same commercial bulk crystal.

Supplementary Table 1. Comparison of optical properties of $MoSe_2$ MMLs and μMLs

		PLQY	PL linewidth	RC linewidth
**MML**	**D/MML/D**	22.6%	8 meV	8 meV
	**MML/SiO_2**	6.2%	>50 meV	26 meV
**μML**	**BN/μML/BN**	28.4%	3 meV	3 meV
	**μML/SiO_2**	13.9%	12 meV	11 meV

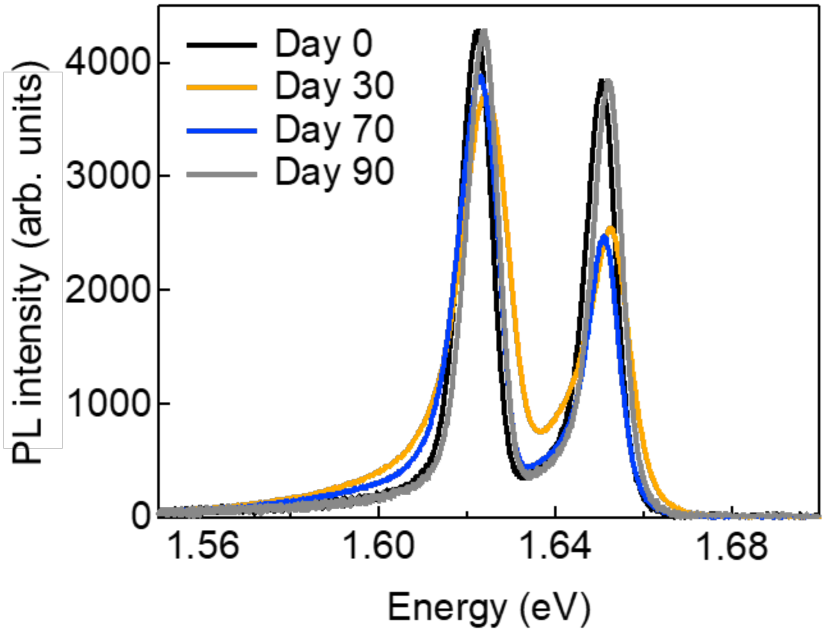

Supplementary Figure 5. Measurement of the stability over time of the molecular encapsulation. PL spectra of D/$MoSe_2$/D MML at 5K after different periods of time.

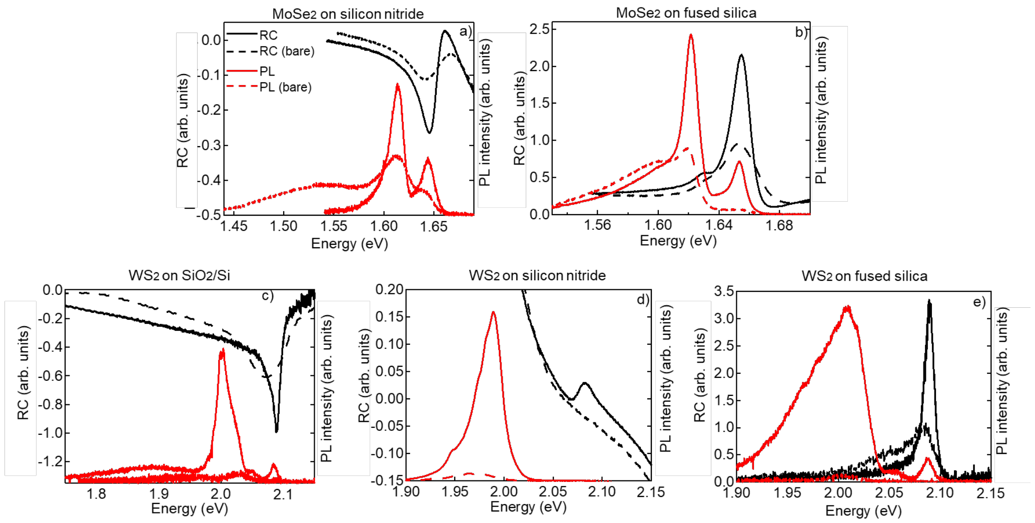

Supplementary Figure 6. Effects of encapsulation for different TMDs and with different substrates. RC (black lines, left axis) and PL (red lines, right axis) spectra at 5K of dodecanol-encapsulated (solid lines) and bare (dashed lines) (a) $MoSe_2$ MML on silicon nitride, (b) $MoSe_2$ MML on fused silica, (c) WS_2 MML on SiO_2/Si, (d) WS_2 MML on silicon nitride, and (e) WS_2 MML on fused silica.

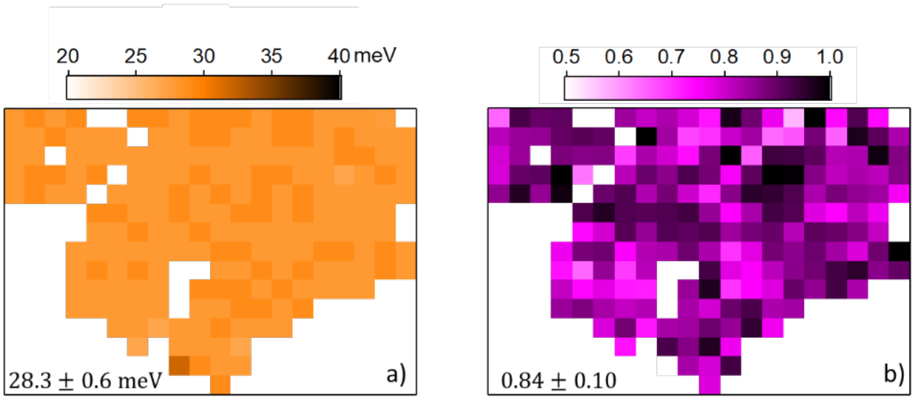

Supplementary Figure 7. Uniformity of trion properties compared to excitons. The mapping of (a) trion binding energy and (b) exciton/trion PL intensity ratio over the D/$MoSe_2$/D MML shown in Figure 2a.

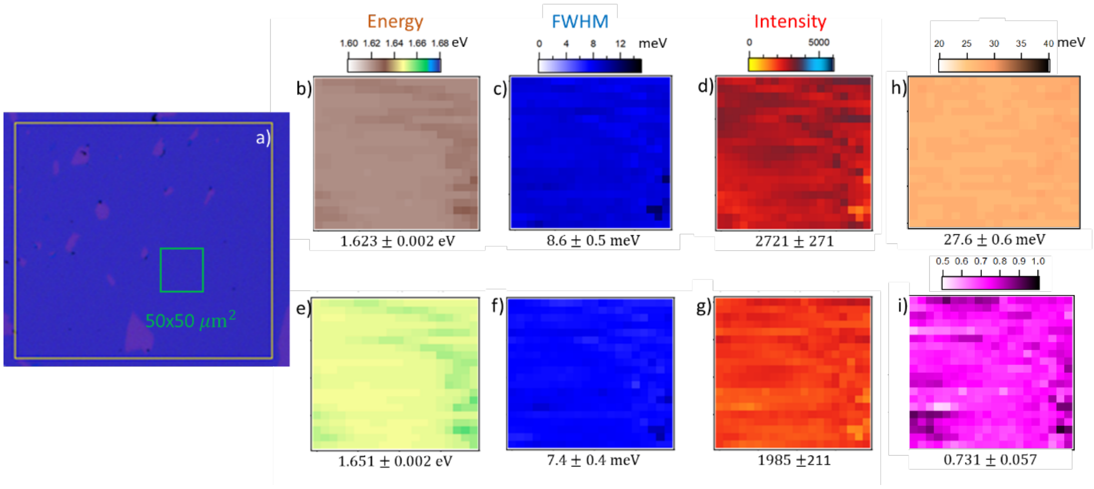

Supplementary Figure 8. Uniformity of PL properties of MML by resolution-limited spatial mapping. (a) Optical microscope image of the sample with the PL mapping region of 50×50 μm^2 marked by the green square. The maps of (b-d) exciton (e-g) trion emission's (b, e) peak energy, (c, f) linewidth, and (d, g) intensity. (h) The map of trion binding energy. (i) The map of exciton/trion PL intensity ratio. The corresponding mean and standard deviation are listed below each map. All data were collected at 5K.

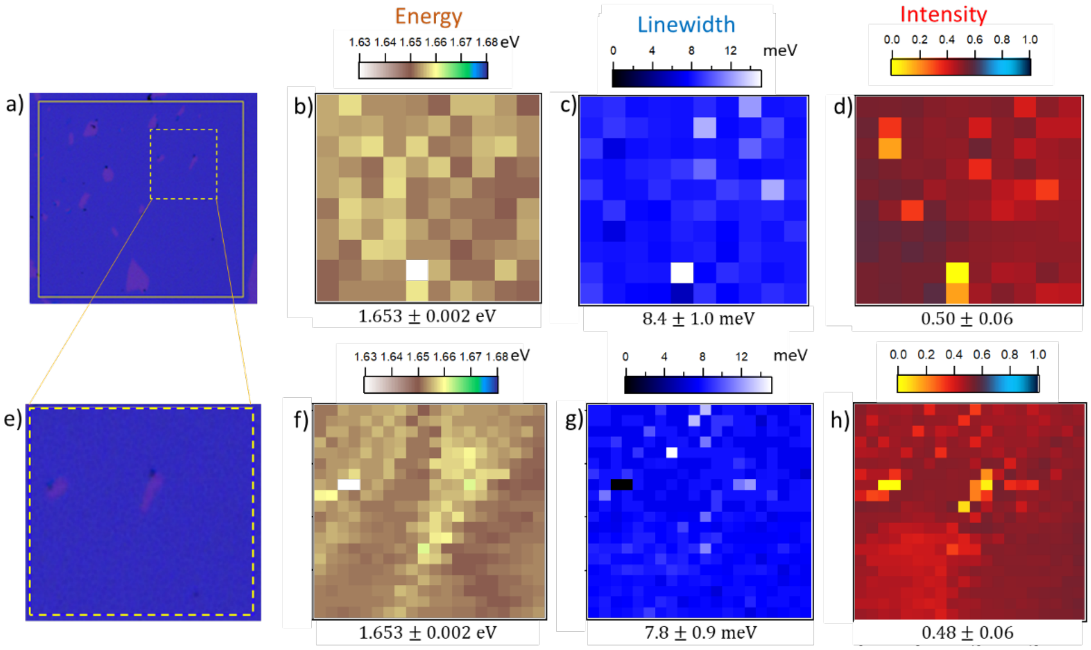

Supplementary Figure 9. Uniformity of RC properties of MML by resolution-limited spatial mapping. RC mapping of two regions at 5K. (a) Optical microscope image of the sample, with the two RC mapping regions marked by the solid and dashed yellow squares, with areas of $230\times230\ \mu m^2$ and $100\times100\ \mu m^2$, respectively. (b-d) Results from RC mapping of the larger area as marked by the solid yellow square, including maps of the exciton (b) energy, (c) linewidth w, and (d) intensity I, where w and I are as defined in Figure 1d. (e) Zoomed in optical microscope image of the smaller RC mapping region as marked by the dashed yellow squares. (f-h) Similar to (b-d) but for the smaller area shown in (e) instead. The corresponding mean and standard deviation are listed below each map.

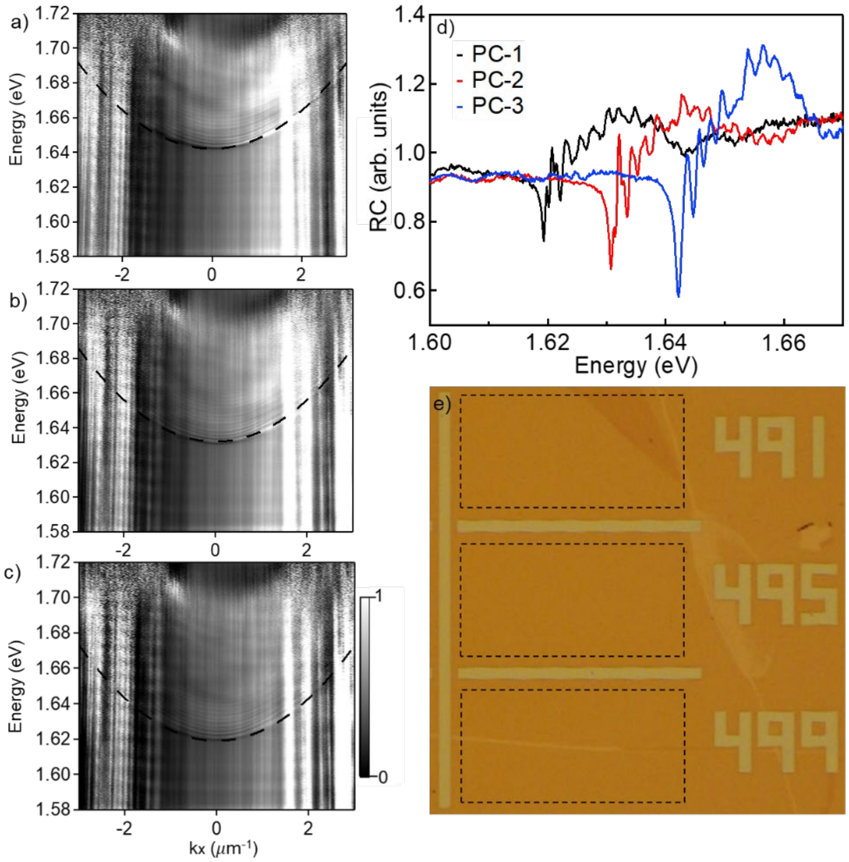

Supplementary Figure 10. Characterizations of photonic crystals (PCs) and the image of D/$MoSe_2$/D MML on PC. The momentum-resolved RC spectra of (a) PC-1, (b) PC-2, and (c) PC-3 at 5K. Black dashed lines are parabolic fits for cavity resonant mode. (d) The RC spectra at k_x=0 for PC-1, PC-2, and PC-3. (e) Optical microscopy image of D/$MoSe_2$/D MML on PC-1 (labeled as 491 because the grating period is 491 nm), PC-2 (labeled as 495), and PC-3 (labeled as 499). Black dashed lines mark the PC region (200×100 μm^2).
